# Identification of differentially expressed genes and pathways for intramuscular fat metabolism between breast and thigh tissues of chickens

**DOI:** 10.1186/s12864-017-4292-3

**Published:** 2018-01-16

**Authors:** Huanxian Cui, Maiqing Zheng, Guiping Zhao, Ranran Liu, Jie Wen

**Affiliations:** 1 Institute of Animal Sciences, Chinese Academy of Agricultural Sciences, Beijing, 100193 China; 2 State Key Laboratory of Animal Nutrition, Beijing, 100193 China

**Keywords:** Microarray, Differentially expressed gene, Regulatory mechanism, Intramuscular fat, Breast and thigh, Chicken

## Abstract

**Background:**

Intramuscular fat (IMF) is one of the important factors influencing meat quality, however, for chickens, the molecular regulatory mechanisms underlying this trait have not yet been clear. In this study, a systematic identification of differentially expressed genes (DEGs) and molecular regulatory mechanism related to IMF metabolism between Beijing-you chicken breast and thigh at 42 and 90 days of age was performed.

**Results:**

IMF contents, Gene Ontology (GO) terms, and Kyoto Encyclopedia of Genes and Genomes (KEGG) pathways were analyzed, The results showed that both IMF contents in breast at 42 and 90 d were significantly lower (*P* < 0.05 or *P* < 0.01) than those in thigh. By microarray, 515 common known DEGs and 36 DEGs related to IMF metabolism were identified between the breast and thigh at 42 and 90 d. Compared to thigh, the expression levels of *PPARG* had significantly down-regulated (*P* < 0.01) in breast, but the expression levels of *RXRA* and *CEBPB* had significantly up-regulated (*P* < 0.01). However, the expression levels of *LPL*, *FABP4*, *THRSP*, *RBP7*, *LDLR*, *FABP3*, *CPT2* and *PPARGC1A* had significantly down-regulated in breast (*P* < 0.01), supporting that *PPARG* and its down-stream genes had the important regulatory function to IMF deposition. In addition, based on of DEGs, KEGG analysis revealed that PPAR signaling pathway and cell junction-related pathways (focal adhesion and ECM-receptor interaction, which play a prominent role in maintaining the integrity of tissues), might contribute to the IMF metabolism in chicken.

**Conclusions:**

Our data had screened the potential candidate genes associated with chicken IMF metabolism, and imply that IMF metabolism in chicken is regulated and mediated not only by related functional genes and PPAR pathway, but also by others involved in cell junctions. These findings establish the groundwork and provide new clues for deciphering the molecular mechanisms underlying IMF deposition in poultry. Further studies at the translational and posttranslational level are now required to validate the genes and pathways identified here.

**Electronic supplementary material:**

The online version of this article (10.1186/s12864-017-4292-3) contains supplementary material, which is available to authorized users.

## Background

Intramuscular fat (IMF) represents deposited lipid in the muscle tissue, which is distributed in the epimysium, perimysium, and endomysium. The certain IMF content in the muscle tissues will not only improve the sensory quality of the meat, but also enhance its flavor, tenderness, and water retention [1–4]. Lipid biosynthesis in chickens mainly occurs in the liver, which is different from pigs, in which lipids are mainly synthesized in the adipose tissue [5]. After synthesis, lipids, in the form of lipoproteins, are transported via blood circulation to target tissues, where the lipoproteins are hydrolyzed by lipoprotein lipase and fatty acids are released for immediate use or deposition [6]. The IMF content depends on the number of adipocytes and the capacity for lipid deposition. The number of adipocytes is decided before birth, and the lipid deposition capability of adipocytes is affected by various factors after birth. Thus, the adipocyte number of an animal is decided prenatally and the deposition of lipids would be completed by increasing the adipocyte volume and weight during subsequent growth [7].

In our previous studies, IMF deposition in thigh was observed to be significantly higher than in breast muscle [8]. IMF deposition is a dynamic process that is regulated comprehensively by hormones and cell factors, including a series of steps such as adipocyte differentiation, and the synthesis, transportation, and decomposition of lipids. The regulation of lipid metabolism is extremely complex because of the interactions of these factors.

In the current work, many studies on the regulation of single gene to IMF had been finished, but few studies on the molecular regulation mechanism of IMF in chickens were performed. In the present study, we used Beijing-you (BJY) chickens, which is a local breed with a rich IMF content, and the Agilent chicken genome array to systematically identify differentially expressed genes (DEGs) related to IMF and explore the molecular regulatory mechanism of IMF through the comparison between breast and thigh tissues at 42 and 90 days of age.

## Results

### Differences in IMF between breast and thigh tissues

**Fig. 1:**
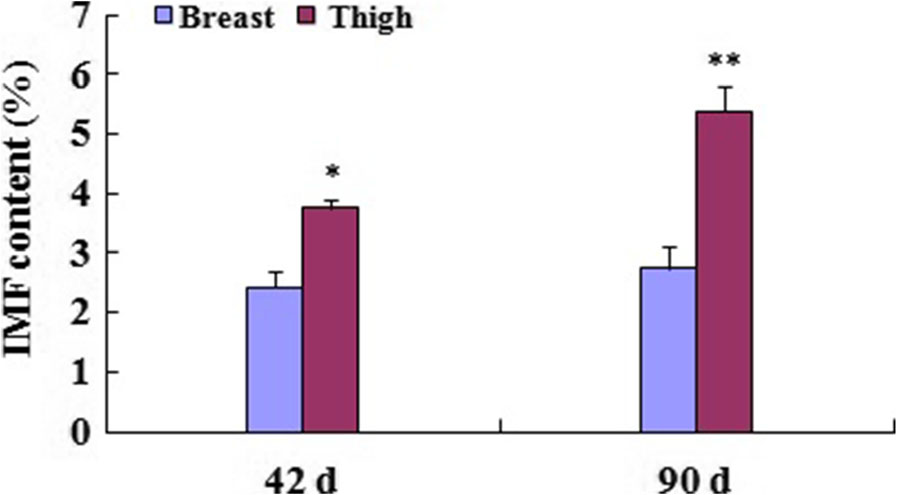
IMF contents in breast and thigh tissues at 42 and 90 d. The IMF contents in thigh were significantly higher (*P <* 0.05, *P <* 0.01, respectively) compared with those in breast at 42 and 90 d. Data are the means ± SD, *n* = 12

The IMF contents in breast and thigh tissues at 42 and 90 d were detected, respectively. The IMF content in thigh tissue (2.43%) was significantly higher (*P <* 0.05) compared with that in breast tissue (3.76%) at 42 day of age. Similarly, the IMF content in thigh tissue (2.74%) was significantly higher (*P <* 0.01) compared with that in breast tissue (5.39%) at 90 day of age (Fig. 1).

### The validation of the microarray and identification of DEGs related to IMF metabolism

**Fig. 2:**
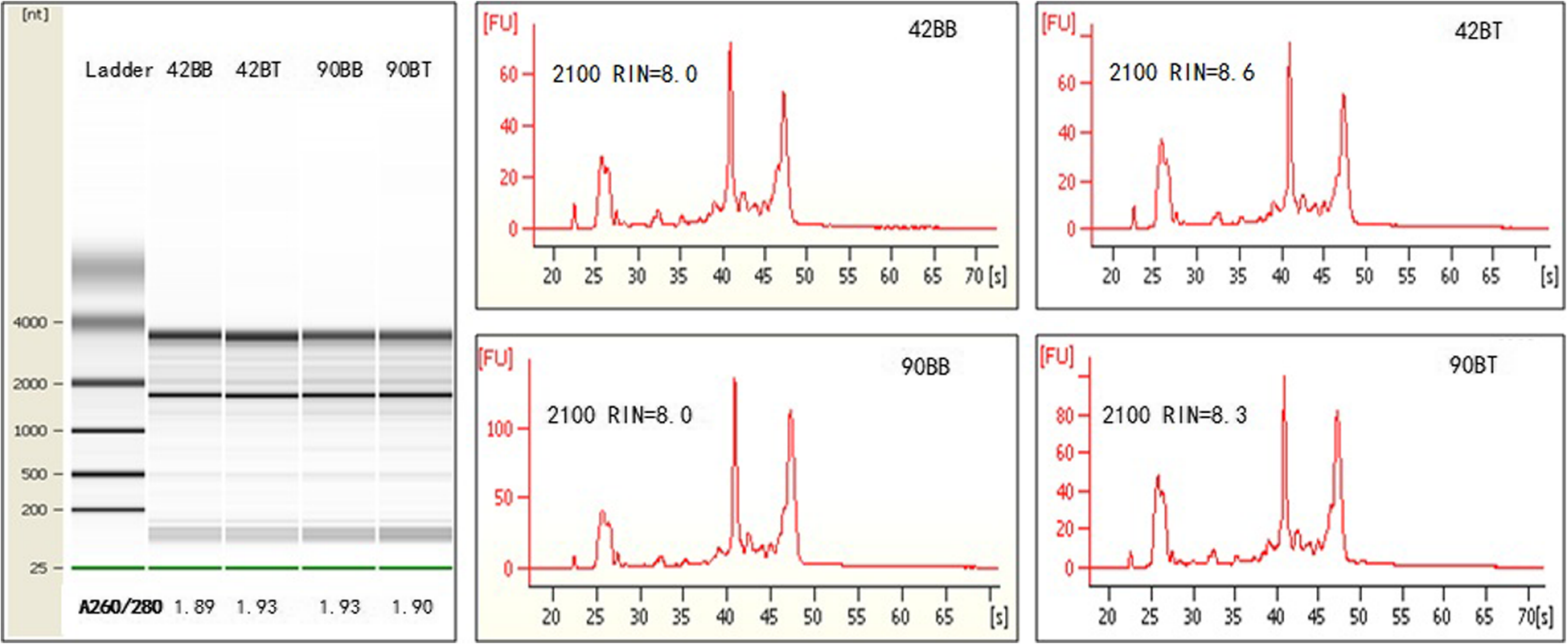
Detection of RNA quality. The total RNAs were separate, their qualities were identified. The results of gel electrophoresis, the ratios of A260/A280 and 2100 RIN showed that the obtained total RNA had the higher acceptance for microarray

**Fig. 3:**
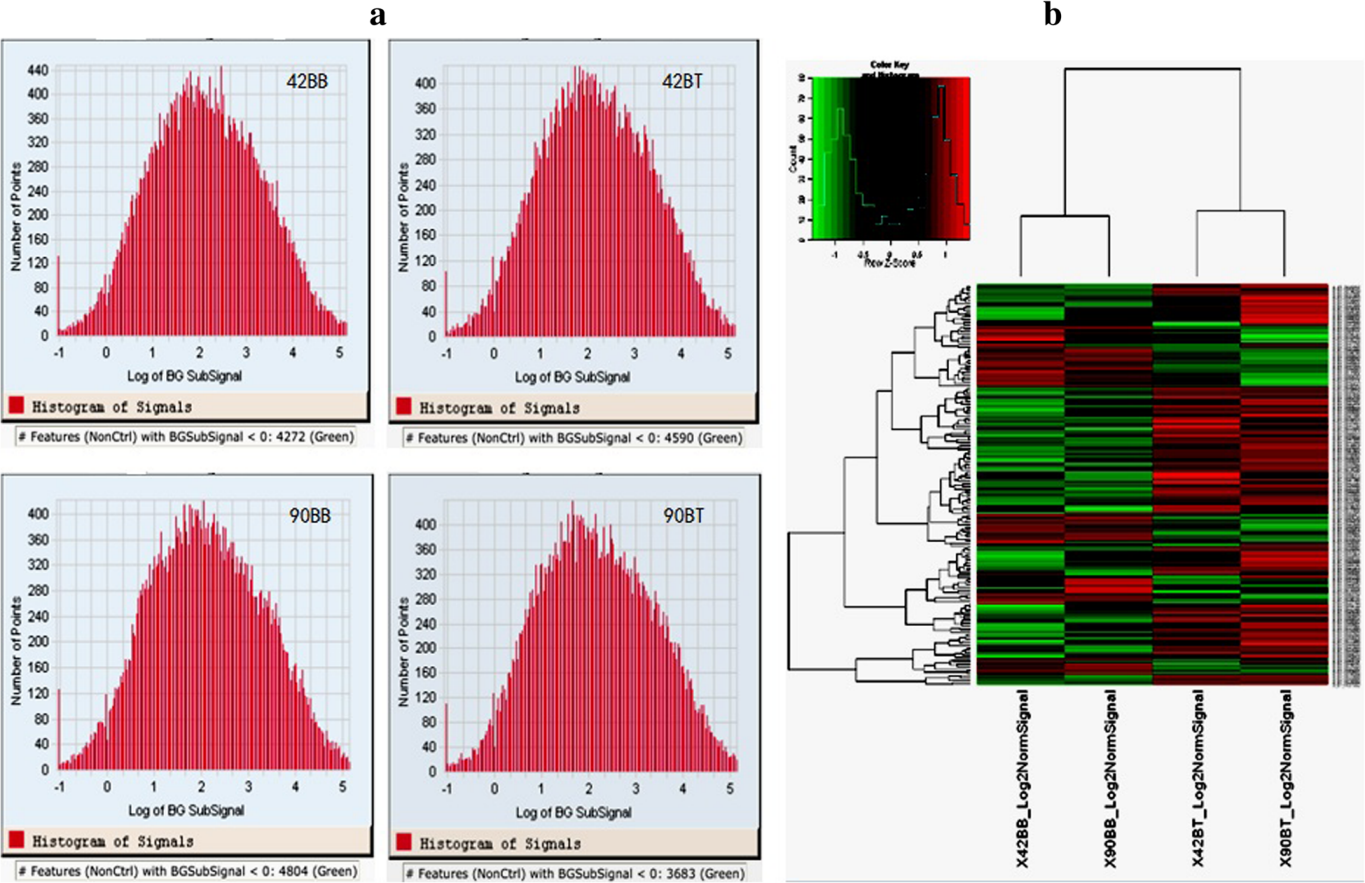
Validation of data in the microarray. **a** the normal distribution test. In each microarray, data was in accordance with normal distribution; **b** cluster analysis of all microarrays. The results showed that the data in the microarrays of chickens at 42 d and 90 d within the same tissue were closely related

Firstly, total RNA were detected to ensure the quality for microarray, and the results showed that the quality of obtained total RNA was satisfying, and could meet the experimental requirements (Fig. 2). The breast was used as the control, and comparisons between the thigh and breast at 42 d and 90 day of age were respectively performed (42BB vs 42BT, and 90BB vs 90BT), respectively. To validate the reliability of the microarray data, the normal distribution analysis was performed in four microarrays, and data of each microarray was in accordance with normal distribution (Fig. 3a), which showed that the microarray data was reliable. The cluster analysis of all microarrays also was performed (Fig. 3b) using the Cluster 3.0 software. The results showed that data in the microarrays of 42 and 90 d within the same tissue were closely related, which also confirmed the reliability of the microarray data. For two comparisons, 515 known genes were detected as DEGs, 290 up-regulated and 225 down-regulated (Additional file 1).

### The identification of DEGs related to IMF metabolism

Using the 515 known DEGs between the thigh and breast at 42 and 90 d, GO analyses were performed, respectively. The common significantly enriched GO terms (*P* < 0.05) in the ontology “Biological Process” of the two comparisons were chosen, including muscle system process, lipid metabolic process, cell cycle, et al. (Additional file 2). On the basis of the enriched GO-terms and 515 common known DEGs, 42 DEGs related to lipid metabolism were screened. Combined with the changes in IMF contents in thigh and breast tissues at 42 and 90 d, certain DEGs related to lipid metabolism were rejected, and the remaining 36 DEGs were considered as DEGs related to IMF metabolism in this study (Additional file 3).

**Fig. 4:**
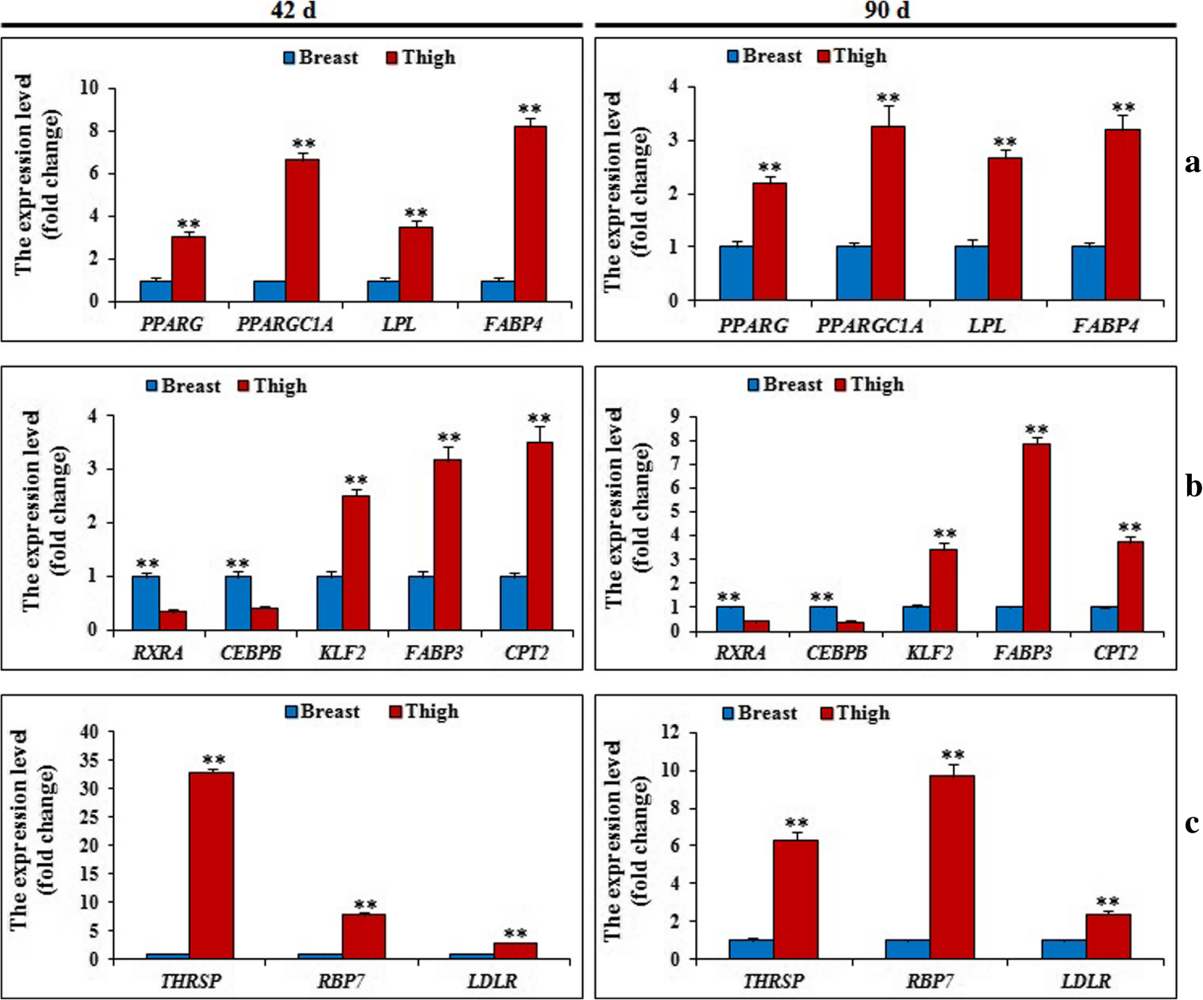
The expression levels of DEGs related to IMF metabolism by q-PCR between breast and thigh tissues at 42d and 90 d. **a** and **c**. Some representative genes involved in accelerating lipid deposition (PPARG, PPARGC1A, LPL, FABP4, THRSP, RBP7 and LDLR) were significantly (*P* <0.01) down-regulated in breast compared with that in thigh at 42 d and 90 d; **b** Other representative genes involved in regulating PPARG (RXRA and CEBPB) were significantly (*P* < 0.01) up-regulated, and fatty acid metabolism (CPT2, FABP3 and KLF2) were significantly (*P* < 0.01) down-regulated in breast compared with that in thigh at 42 d and 90 d. Data are the means ± SD, *n* = 6

Among them, some representative genes related to lipid metabolism were found, and the verifications of their mRNA levels by Q-PCR were performed (Fig. 4). Though the fold changes of *PPARG* were 2.45 and 1.92 (Additional file 3), it also was detected by Q-PCR for the importance in lipid metabolism. Compared to thigh, the expression levels of *PPARG* had significantly down-regulated (*P* < 0.01) in breast. However, the expression of *RXRA* and *CEBPB*, had significantly up-regulated (*P* < 0.01), contradicting with previous reports. Meanwhile, the mRNA levels of *KLF2* and *PPARGC1A* had significantly down-regulated (*P* < 0.01). However, as the target genes of PPARG, the expression levels of *FABP4, LPL, CPT2* and *FABP3* had significantly down-regulated in breast (*P* < 0.01). In addition, *THRSP*, *RBP7* and *LDLR*, which accelerate the lipid biosynthesis, also had significantly down-regulated in breast (*P* < 0.01).

**Fig. 5:**
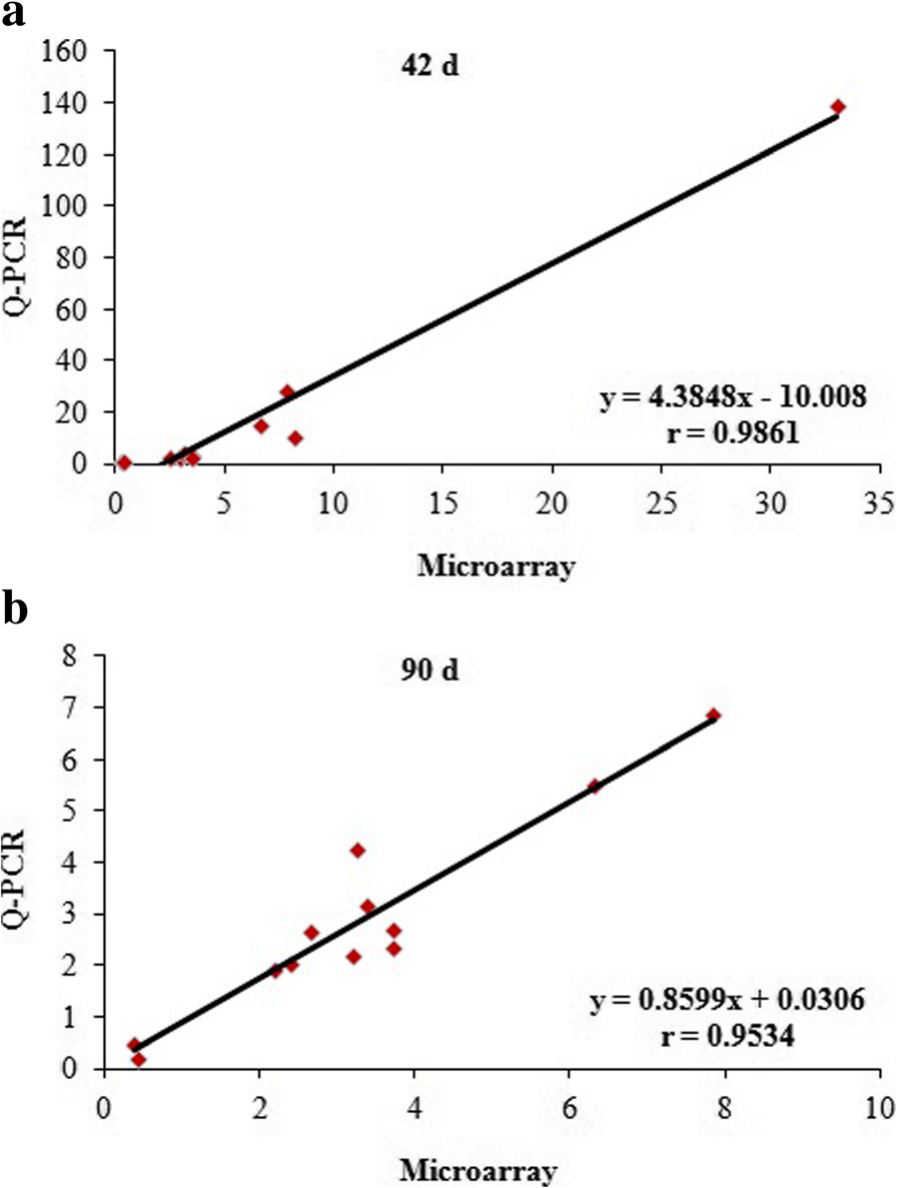
Technical validation of microarray results using q-PCR correlation. **a** and **b**. The r = 0.9861 (42 d,* P* < 0.01) (**a**) and r = 0.9534 (90 d, *P* < 0.01) (**b**) indicate that the Spearman Rank Correlation between breast and thigh were positive. This indicated that the q-PCR fold changes were in complete correspondence with the microarray data in two days of age. Data are the means ± SD, *n* = 12

To deeply confirm results from the microarrays, the fold changes of the above 12 genes in microarray and q-PCR were related using Spearman rank correlation. As shown in Fig. 5, fold-changes in gene expression between breast and thigh by two methods were correlated at 42 d (*r* = 0.9861, *P* < 0.01) and 90 d (*r* = 0.9534, *P* < 0.01). This result also showed the consistency between the results of DEGs by q-PCR and the microarray analyses, and highly confirmed the reliability of the microarray data.

### The analysis of pathways on IMF metabolism

KEGG pathway analysis for the 515 common known DEGs from the two comparisons was performed, and the enriched (q-value *<*0.05) KEGG pathways were screened (Table 1). As expected, PPAR signaling pathway were found, and other significant pathways included focal adhesion, ECM-receptor interaction, et al. The DEGs involved in these pathways were also screened, and 10, 18 and 9 DEGs were involved in the PPAR signaling pathway, focal adhesion and ECM-receptor interaction, respectively.

**Table 1:** The enriched (*P <* 0.05) KEGG pathways in microarray

Pathways	Hits	Total	*P*-value
PPAR signaling pathway	10	59	0.000972
Focal adhesion	18	184	5.11E-05
ECM-receptor interaction	9	73	0.001877
Valine. leucine and isoleucine degradation	5	44	0.048259
Propanoate metabolism	5	25	0.007102
Pyruvate metabolism	6	34	0.008396

For two pathways (focal adhesion and ECM-receptor interaction), 5 commom DEGs (*COL5A2*, *COL6A2*, *COL6A3*, *RELN* and *SPP1*) were found (Additional file 4). In addition, the DEGs related to IMF metabolism were also respectively found in Focal adhesion (*CAV1*) and ECM-receptor interaction (*CD36*).

**Fig. 6:**
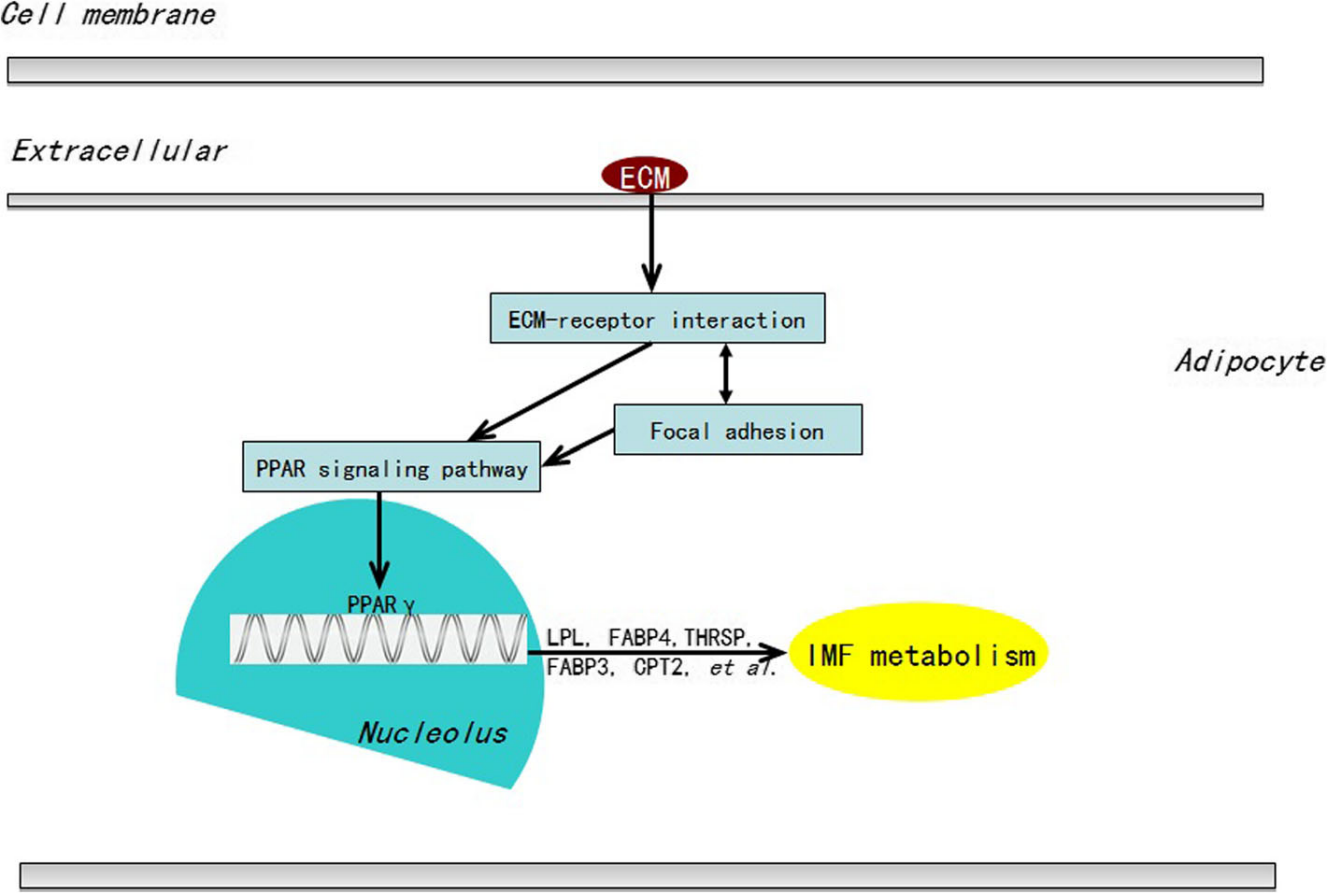
The potentially regulatory network of IMF metabolism according to DEGs the enriched KEGG pathways. This network is involved in IMF metabolism including PPAR signaling pathway and cell junction (focal adhesion, ECM-receptor interaction)

Overall consideration to the results of the IMF contents, DEGs related to lipid metabolism and the KEGG pathways in this study, we then constructed the hypothetical and potential regulatory mechanism for IMF metabolism using the biological and gene function information in NCBI Entrez (Fig. 6).

## Discussion

Deposited lipids in chickens include mainly abdominal fat, subcutaneous fat and IMF, and most studies have been performed on abdominal fat. The expressions of certain genes, involved in lipid deposition, have been analyzed in chickens [9–12]. Some research had been finished to systematically analyze the DEGs and regulatory mechanism in abdominal fat or IMF of breast using a cDNA microarray [13, 14]. However, few studies have systematically analyzed the gene expression profiles related to IMF in breast and thigh of chickens. In this study, the present objective was to identify global genes and pathways affecting chicken IMF metabolism to explore the regulatory mechanism of IMF between breast and thigh tissues of BJY chickens at 42 d and 90 d by cDNA microarray.

### cDNA array analysis

After obtained the high quality total RNA, chicken microarrays were employed, each using pooled RNA samples (*n* = 6 birds, within each of two tissues and at 2 ages; 4 arrays in all). Such a pooling strategy can dramatically improve accuracy when only one array is available in each biological condition [15]. To validate the results from the microarray, a normal distribution test and cluster analysis of all microarrays were performed. In each microarray, data was in accord with the normal distribution test. Moreover, the cluster analysis showed a close relationship between the data of two microarrays at 42 d and 90 d among thigh tissue and breast tissue, respectively. This result correlated with the known physiological development of chickens and confirmed the reliability of the microarray data. In this study, 12 DEGs were selected from the 36 common DEGs related to IMF metabolism to deeply validate the results, more than 52 tests were done by q-PCR, and fold-changes in gene expression between the two methods were highly correlated at 42 d and 90 d in chickens. These results further confirmed the reliability of the microarray data.

### The genes related to lipid biosynthesis are responsible for IMF deposition

According to GO-term analysis on basis of 515 common known DEGs, 36 DEGs related to IMF metabolism were shared by thigh and breast at 2 ages. Among them, PPARG plays an important role in regulating adipocyte differentiation and lipid deposition [16–18], PPARGC1A is a co-activator of PPARG [19, 20], and their expression had significantly down-regulated in breast (*P <* 0.01) compared to those in thigh at 42 and 90 d. It was misleading that the mRNA levels of RXRA and CEBPB, as the cofactor of PPARG [21, 22], had significantly up-regulated in breast (*P <* 0.01). In addition, the expression of KLF2, which would negatively regulate *PPARG* expression [23], also had significantly decreased in breast (*P <* 0.01). However, as the targets of PPARG [24], the expression levels of *LPL* and *FABP4* had significantly down-regulated in breast (*P <* 0.01) compared to those in thigh at 42 and 90 d in this study. LPL is the rate-limiting step in the hydrolysis of triglyceride from circulating chylomicrons and very low density lipoprotein [25–27]. FABP4 has been considered as a major cytoplasmic protein related to glucose and lipid metabolic functions [28, 29]. These results strongly supported the view of down-regulation of *PPARG* in breast, revealing that thigh tissue had the stronger lipid biosynthesis compared to breast tissue.

Similarly, the expression levels of *THRSP*, *RBP7*, *PLIN* and *LDLR* had significantly down-regulated in breast (*P <* 0.01) than in thigh at 42 and 90 d. As the previous reported, THRSP encodes a small acidic protein that is implicated as a transcription factor involved in control of lipogenic enzymes [30], RBP7 had the important function in regulating the lipid deposition [31], LDLR is a single-chain transmembrane glycoprotein that regulates the plasma cholesterol level [32–34]. So it was revealed that THRSP, PLIN, and LDLR would participate in the regulation of IMF deposition in this study.

On the other hand, FABP3 and CPT2 would participate in the processes of lipolysis process. FABP3 is involved in fatty acid transport from cell membrane to the intracellular sites of fatty acid utilization and is mainly expressed in cardiac and skeletal muscle [35]. CPT2 is one of the two proteins of the carnitine transport system [36], and CPT2 deficiency is an inherited disorder of long-chain fatty acid oxidation [37, 38]. Their expression levels of these genes had significantly down-regulated in thigh (*P <* 0.01) compared to those in breast at 42 and 90 d. These results revealed that thigh tissue also had the stronger energy supply compared to breast tissue.

The regulation of IMF is possibly a function of complex pathway interactions involving muscle, fat and connective tissue [39]. Combined the data of IMF in breast and thigh tissues, genes related to lipid biosynthesis were dominant than other genes related to lipolysis, and responsible for the more IMF content in thigh tissue.

### The pathways of PPAR and cell junctions participated in the regulation of IMF deposition in chicken

KEGG pathway analysis was used to explore the regulatory network underlying chicken IMF deposition. As expected, the well-known PPAR pathway was found. According to the PPAR signaling pathway (gga03320) and the known information, 10 DEGs (*PPARG, RXRA, ACSF3, ACSS2, LPL, CD36, CPT2, CYP27A1, FABP3, FABP4*) involved in PPAR signaling pathway here were screened, which have been proven to be functional in lipid metabolism, such as FABP3, FABP4, CD36, LPL.

Of special interest, two pathways (focal adhesions and ECM-receptor interaction) also were enriched, and 5 common known DEGs here were shared by focal adhesions and ECM-receptor interaction. It was revealed that these two pathways had the points for the interaction. Moreover, CAV1 and CD36, which screened as the DEGs in this study, were respectively found in focal adhesions and ECM-receptor interaction. CD36, facilitated the inward transport of fatty acids [40, 41], was involved in ECM-receptor interaction [42, 43]. In support of this, previous studies have shown that changes in cytoskeletal organization and its contacts with the ECM are essential in the morphogenesis of fibroblastic preadipocytes to rounded, mature adipocytes [44]. Caveolins (CAV) are essential components of caveolae, CAV proteins bind cholesterol, and CAV’s ability to move between cellular compartments helps control intracellular cholesterol fluxes [45, 46].

Taken together, the results revealed that cell junctions (focal adhesions and ECM-receptor interaction) might regulate IMF accumulation by associated with the PPAR signaling pathway during chicken development, and the proposed molecular regulatory network affecting IMF metabolism during chicken development is presented in Fig. 6. This is partially consistent with our previous study [14].

## Conclusions

In this study, with the aim of identifying the candidate genes and new pathways related to IMF metabolism for regulatory mechanism between breast and thigh in chicken, it was found that *PPARG*, *LPL*, *FABP4*, *THRSP*, *RBP7*, *PLIN* and *LDLR* would be responsible for the IMF deposition in chicken. In addition, IMF deposition in chickens is regulated and mediated not only by genes related to lipid metabolism and PPAR signaling pathway, but also by others involved in cell junctions with the function in maintaining the integrity of tissues and signal transduction.

These findings establish the groundwork and provide new clues for deciphering the molecular mechanisms underlying IMF metabolism in chicken. Further studies at the translational and posttranslational level are now required to validate the genes and pathways identified here.

## Methods

### Animals and sample collection

All 36 Beijing-You chickens (BJY, the Institute of Animal Sciences, Chinese Academy of Agricultural Sciences, Beijing, China) with the same genetic background, entered the experiment at the same time and were randomly distributed into 3 replicate groups, each of 12 birds. Birds were reared in stair-step caging of an environmentally controlled room under continuous lighting using standard conditions of temperature, humidity and ventilation. Feed and water were provided ad libitum during the experiment. Diets were formulated according to Nutrient Requirements of Yellow-feathered Broiler (NY/T 33–2004, China).

At the sampling times of 42 and 90 d, 12 birds with similar weight from each day were selected and killed by stunning and exsanguination after fasting for 12 h. Samples of the pectoralis major muscle (BB) and deboned thigh muscle (BT) were snap frozen in liquid nitrogen and stored at −80 °C, others were collected and stored at −20 °C.

### Measurement of IMF content

Two grams of each sample from 12 birds, after eliminating obvious fat, were minced thoroughly after being thawed and dried in two 10–12 h stages (at 65 °C and 105 °C, respectively), followed by cooling in a desiccator for at least 30 min. The IMF contents in the pectoralis major muscle and deboned thigh muscle were measured by the Soxhlet method [47, 48], using anhydrous ether as the solvent, and were expressed as percentages, on the basis of dry tissue weight.

### RNA extraction and identification

The tissue samples (breast or thigh) from 6 birds at each day of age, which had the significant difference of IMF between breast tissue or thigh tissue were selected. The total RNA was isolated using the Trizol reagent (Invitrogen, USA) according to the manufacturer’s instructions. After the quality verification on gel electrophoresis, A260/A280 and RNA integrity number (RIN), RNA was dissolved with 1 μg/μl concentration and six RNA samples from thigh or breast tissues at 42 and 90 d with equal concentrations were pooled for every microarray analyses.

### Microarray hybridization and analysis

Agilent Gene Chip microarray with 42,034 probe sets (ID: 015068) was finished by Shanghai Biotechnology Corporation (Shanghai, China). Array scanning and data extraction were carried out following the manufacturer’s standard protocol.

The normal distribution of signals plot in every chip was provided. Clustering was performed using uncentered Pearson correlations and the average linkage cluster 3.0, and was displayed in TreeView. Normalized fluorescence intensity values of each dye-swapped experiment were averaged separately for sample and reference channels. Thereafter, for each probe, averaged sample and reference fluorescence values were log2-transformed. Average linkage hierarchical clustering was performed using the Euclidian metric. In the generated heat maps, the colors of the features (probes) were determined by log2 transformation (sample/reference).

### Identification of differentially expressed genes (DEGs)

The distribution of expressed genes was analyzed by JMP4.0, according to their expression levels. If the flag of a gene was assigned as “P” by the scanner according to the data normalization and Agilent Microarray Suite 4.0 software results, it would be considered as expressed transcripts. The expression value of each probe set was normalized and calibrated using the robust multi-array average (RMA) method.

The data of four groups (42BB, 42BT, 90BB and 90BT) were divided into two comparisons (42BB vs 42BT and 90BB vs 90BT). Differentially expressed probe sets were identified using a cutoff of fold-change ≥2.0 in both comparisons of 42 and 90 d between thigh and breast tissues. Gene Ontology (GO) enrichment analysis was performed for DEGs related to IMF metabolism at each time point using the GOEAST software toolkit [49]. The significance level of GO term enrichment was set as a false discovery (FDR)-adjusted *p*-value smaller than 0.1, by the Benjamini-Yekutieli method [50]. According to the results of GO enrichment analysis, DEGs related to IMF metabolism were screened.

### Real-time quantitative PCR (Q-PCR)

To avoid amplification of contaminating genomic DNA, all primers were placed at or just outside of exon/exon junctions and listed in Additional file 5. Q-PCR was employed using SYBR Green PCR Master Mix (ABI) in the ABI Prism 7500 System under the following conditions: 95 °C for 10 min for 1 cycle, followed by 40 cycles at 95 °C for 15 s, and then at 60 °C for 45 s. Q-PCR was performed in triplicate with standard deviations of threshold cycle (CT) values not exceeding 0.5.

### The Kyoto encyclopedia of genes and genomes (KEGG) pathway analysis

KEGG pathway [51–53] information was used in this analysis. Probe set IDs of each category were first mapped to NCBI Entrez gene IDs according to the Agilent Chicken Array annotation file, and then mapped to KEGG gene IDs according to the KEGG gene cross-reference file. Pathways that were significantly enriched for DEGs were identified using a hypergeometric test in the R packages *(P* < 0.1, FDR adjusted). Pathways with fewer than three known chicken genes were discarded. Graphical pathway maps were downloaded from the KEGG FTP server, and DEGs were then highlighted in them, according to the coordinate description in XML files at the KEGG FTP server, using Perl GD, XML::Parser, and XML::LibXML modules.

### Statistical analyses

Statistical differences between groups were evaluated using the Student’s *t*-test. All computations were made using one-way ANOVA and Statistical Analysis Systems software (Version 8.2, SAS Institute, Cary, NC, USA, 2001). *P* < 0.05 (*) or *P* < 0.01 (**) was considered significant. Data are represented as mean ± SD.

## Additional files

Additional file 1: The DEGs using a cutoff of fold-change ≥1.5 in both comparisons of 42 and 90 d between thigh and breast tissues. (XLS 154 kb)

Additional file 2: The biological process of enriched GO terms based on 515 DEGs. (XLS 516 kb)

Additional file 3: The DEGs related to lipid metabolism in both comparisons of 42 and 90 d between thigh and breast tissues. (XLS 55 kb)

Additional file 4: The common DEGs involved in two pathways (ECM-receptor interaction and Focal adhesion) in this study. (XLS 40 kb)

Additional file 5: The specific primers for q-PCR in this study. (XLS 57 kb)

## Abbreviations

ACSF3: Acyl-CoA synthetase family 3
ACSS2: Acyl-CoA synthetase short-chain family member 2
BJY: Beijing-you
C/EBPB: CCAAT/enhancer binding protein beta
CAV: Caveolin
CD36: CD36 molecule
COL: Collagen
CPT2: Carnitine palmitoyltransferase II
DEG: Differentially expressed gene
ECM: Extracellular matrixc
FABP: Fatty acid binding protein
GO: Gene ontology
IMF: Intramusclar fat
KEGG: Kyoto Encyclopedia of Genes and Genomes
KLF: Kruppel-like transcription factors
LDLR: Low density lipoprotein receptor
LPL: Lipoprotein lipase
PLIN: Perilipin
PPARG: Peroxisome proliferator-activated receptor gamma
PPARGC1A: Peroxisome proliferator activated receptor, gamma, coactivator 1 alpha
Q-PCR: Real-time Quantitative PCR
RBP7: Retinol binding protein 7, cellular
RELN: Reelin
RXRA: Retinoic acid receptor -alpha
SPP1: Secreted phosphoprotein 1)
THRSP: Thyroid hormone responsive (SPOT14 homolog)
